# Early decompressive hemicraniectomy combined with mild hypothermia treatment for malignant middle cerebral artery infarction

**DOI:** 10.3389/fneur.2026.1801932

**Published:** 2026-05-20

**Authors:** Junhui Chen, Jiaming Cao, Xiaoyan Feng, Yinchuan Liu, Chunlei Zhang, Yunna Tao, Hui Lan, Yan Wu, Yuhai Wang, Zhonghua Shi

**Affiliations:** 1 Department of Neurosurgery, 904th Hospital of Joint Logistic Support Force of PLA, Wuxi Clinical College of Anhui Medical University, Wuxi, China; 2 Department of Neurosurgery, Changsha Taihe Hospital, Changsha, China; 3 Department of Neurosurgery, Affiliated Huishan Hospital of Xinglin College, Nantong University, Wuxi Huishan District People's Hospital, Wuxi, China

**Keywords:** decompressive hemicraniectomy, malignant MCA infarction, mild hypothermia, mRS, stroke

## Abstract

**Background:**

Decompressive hemicraniectomy (DHC) and mild hypothermia (MH) have been recommended as lifesaving management strategies for patients with uncontrolled intracranial pressure (ICP), such as malignant middle cerebral artery (MCA) infarction. However, relevant clinical studies to substantiate this are lacking.

**Methods:**

A retrospective comparative cohort conducted at three centers between January 2017 and January 2023 enrolled 210 patients with malignant MCA infarction were treated with DHC, 119 patients who underwent DHC + MH, and another 91 patients who underwent DHC surgery alone. Information was obtained regarding patient characteristics during follow-up. Six-month clinical outcomes (6-month mRS score) and 30-day all-cause mortality were analyzed. We also explored differences in post-operative complications between the two groups.

**Results:**

Both groups had comparable baseline characteristics, with no significant differences observed. Lower NIHSS scores (*OR* = 0.179, 95% CI: 1.070–1.336, *P* = 0.002), and early DHC (*OR* = 0.171, 95% CI: 1.113–1.264, *P* = 0.000) may be more beneficial for the patient's prognosis. At 6 months, the DHC + MH group had better mRS scores (*P* = 0.025) and 30-day all-cause mortality (17.6 vs. 29.7%, *P* = 0.04). There were significant differences in shivering (46.2% vs. 6.6%, *P* = 0.000), bradycardia (68.1% vs. 38.5%, *P* = 0.000), electrolyte disturbance (65.5% vs. 38.5%, *P* = 0.002), and acidosis (35.5% vs. 18.7%, *P* = 0.008), and the occurrence of these complications was significantly greater in the DHC + MH group than in the DHC alone group. There was no difference in delayed intracranial hematomas, pneumonia, acute kidney injury (AKI) or coagulation disorders.

**Conclusions:**

In this retrospective multi-center cohort, mild hypothermia during decompressive hemicraniectomy was associated with favorable unadjusted outcomes. Given non-randomized allocation and potential confounding, these findings are hypothesis-generating and require confirmation with adjusted analyses and prospective evaluation. The procedure needs to be validated in larger, multicenter, prospective, randomized controlled trials.

## Introduction

Stroke continues to be the primary cause of mortality in developing nations (1) and is responsible for approximately 5% of disability-adjusted life-years and 10% of deaths worldwide (2, 3). The global lifetime risk of having a stroke from age 25 onward is estimated at 24.9%, with a particularly high risk in East Asia of 38.8% (1). Approximately 3–10% of individuals with malignant acute ischemic stroke develop cerebral infarctions that occupy space caused by full blockage of the MCA, usually leading to uncontrollable, progressive brain edema and intracranial pressure (ICP) (4), which may lead to transtentorial herniation and ultimately death (4, 5), with a mortality rate ranging from 66%−80% (6, 7). Therapeutic methods are very rare when faced with life-threatening conditions. Decompressive hemicraniectomy (DHC) is one of the very important recommended methods because it is associated with favorable outcomes (8, 9). Recently, some randomized controlled trials (RCTs) have demonstrated that DHC surgery within 48 h of malignant middle cerebral artery (MCA) infarction onset can reduce mortality and improve outcomes (10–12). However, many survivors do not regain independence in activities of daily living with severe disability or coma (13–15), and 59% of the initial group has died after a period of two years (16). Hence, there are many controversies regarding DHC treatment for malignant MCA infarction; however, its value is still unclear, and some resistance to its application still exists.

Another important therapeutic method is mild hypothermia, which has been confirmed by many animal experiments (17–19). Previous human studies have shown a beneficial effect of hypothermia on malignant brain infarction (20, 21). However, many studies have indicated that hypothermia leads to many related complications and an increased risk of side effects (22), and the effective results have come from poor evidence, with no evidence from randomized trials of hypothermia as a treatment for malignant MCA infarction (5). Els (23) reported that hemicraniectomy combined with mild hypothermia was associated with a better prognosis, with fewer adverse reactions and side effects, as well as better functional recovery. Our previous studies also demonstrated that DHC + MH was safe and effective for treating patients with malignant ICP after severe traumatic brain injury (24, 25). However, no multicenter studies with larger numbers of patients and long-term outcome evaluations have been conducted.

Consequently, the purpose of this study was to examine the mortality rate at 30 days and the outcome at 6 months from the use of early DHC + MH treatment for intracranial hypertension due to malignant MCA ischemic infarction as a lifesaving procedure.

## Methods

### Patient population

**Figure 1:**
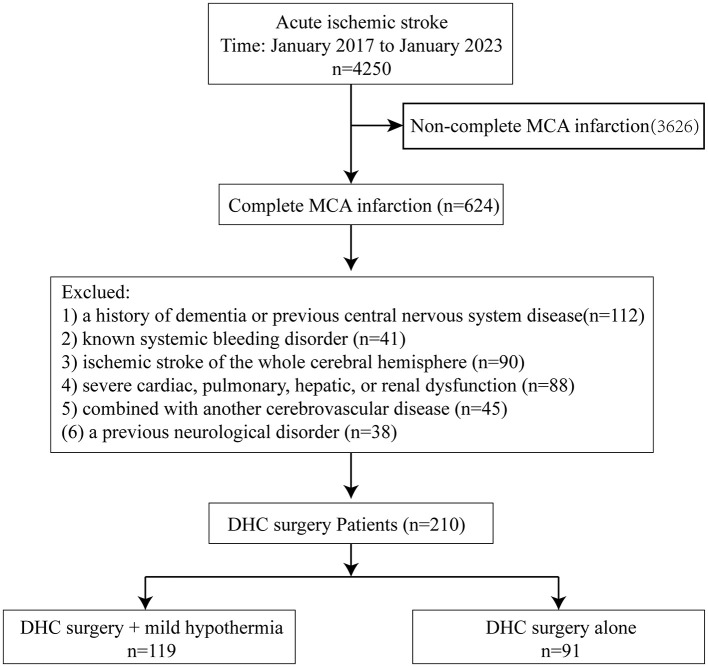
Trial profile.

A retrospective comparative cohort conducted at three centers (904th Hospital of Joint Logistic Support Force of PLA, Wuxi Huishan District People's Hospital, and Changsha Taihe Hospital) between January 2017 and January 2023, 4,250 patients with acute ischemic stroke were admitted to our three clinical institutions, including 624 (14.7%) patients who were diagnosed with complete middle cerebral artery infarction, as determined by computerized tomography angiography (CTA) or digital subtraction angiography (DSA) scans. We enrolled 210 (4.9%) patients who underwent DHC surgery, 119 (56.7%) of 210 patients who underwent DHC + MH, and another 91 (43.3%) patients who underwent DHC surgery alone. Allocation protocol-driven, based on clinician discretion, influenced by patient/family preferences, and financial situation. There were 116 (55.2%) men and 94 (44.8%) women, with an average age of 65 years (range, 34–89 years). All admitted patients were assessed via the National Institutes of Health Stroke Scale (NIHSS) within 2 h and the Glasgow Coma Scale (GCS) before DHC (Figure 1, Table 1). The surgical indications for DHC are as follows: signs on CT of an infarct of at least 50% of the MCA territory, with or without additional infarction in the territory of the anterior or posterior cerebral artery on the same side, or infarct volume >145 cm^3^ as shown on diffusion-weighted MRI; Clinical deficits suggestive of infarction in the territory of the MCA with a score on the National Institutes of Health stroke scale (NIHSS) >15, etc (26).

**Table 1:** Patient characteristics.

Characteristics	Value
Sex, *n*, 100%	
Male	116 (55.2%)
Female	94 (44.8%)
Mean Age (years, mean ± SD)	65 ± 12.2
Admitted NIHSS total score (mean ± SD)	29.5 ± 11.0
GCS score before DHC (mean ± SD)	7.6 ± 3.0
Comorbidities, *n*, 100%	
Hypertension	132 (62.9%)
Hyperlipidemia	99 (47.1%)
Smoking	75 (35.7%)
Diabetes	65 (31.0%)
Atrial fibrillation	46 (21.9%)
Prior stroke(s)	38 (18.1%)
Acute intervention, *n*, 100%	
Intravenous thrombolysis	71 (33.8%)
Mechanical thrombectomy	40 (19.0%)
Admitted to DHC (h, mean ± SD)	29.7 ± 14.2
MLS (mm, mean ± SD)	10.5 ± 4.6
Pupillary dilation pre-operation	67 (31.9%)

NIHSS, National Institutes of Health Stroke Scale; GCS, Glasgow Coma Scale; DHC, decompressive hemicraniectomy; MH, mild hypothermia; MLS, peak midline shift; SD, standard deviation.

### Inclusion criteria and exclusion criteria

The inclusion criteria and exclusion criteria were the same as those reported previously (26, 27). A total of 210 patients with space-occupying malignant MCA infarction were found in three hospitals throughout the study period. The inclusion criteria were as follows: 1) patients who received DHC treatment within 48 h after malignant MCA infarction, and 2) patients whose ischemic alterations on CT scans affected two-thirds or more of the MCA area, accompanied by the development of space-occupying edema during clinical worsening. The exclusion criteria were as follows: 1) a previous diagnosis of dementia or earlier central nervous system illness; 2) a recognized systemic bleeding condition; 3) an ischemic stroke affecting the entire cerebral hemisphere; 4) significant dysfunction of the heart, lungs, liver, or kidneys; 5) combined with another cerebrovascular disease (including moyamoya and vascular malformation); and 6) a previous neurological disorder.

### Clinical outcomes and 30-day all-cause mortality

The neurologic outcome following surgery was determined by measuring the modified Rankin score (mRS) 6 months later via either a phone call or an in-person meeting with two neurosurgeons. If there is any dispute regarding the follow-up results, seek a third reviewer ruling. The outcome assessors were blinded in the present study. The primary functional endpoint is mRS at 6 months, the outcome was dichotomized as good (mRS score 0–2) or poor (mRS score 3–6), and deaths were coded mRS = 6. In the present study, the primary clinical prognosis was the mRS score, and the secondary clinical prognosis was 30-day all-cause mortality.

### Surgical technique

DHC was performed as previously described (23, 26, 28). Surgical timing was important, and early surgery refers to the DHC surgery performed within 48 h of the onset of the disease. The DHC surgical method involves removing a large bone flap measuring 15 cm by 10 cm, which includes the frontal, parietal, temporal, and parts of the occipital squama, to access the floor of the middle cerebral fossa. After opening the dura, a tailored biconvex dural patch made from lyophilized cadaver dura or homologous temporal fascia was positioned in the incision. The size of the dural patch varied, but we usually used patches that were 15–20 cm in length and 2.5–3.5 cm in width. During the operation, if the brain tissue was found to be 1–2 cm above the bone window and had poor pulsation, an infarction core resection procedure was performed. Using the neuro-navigation system for real-time positioning of the ischemic penumbra, the infarction core and the corticospinal tract, the infarction core was removed under a microscope. During the operation, efforts were made to avoid damaging the cortical functional areas and important blood vessels. Maintaining stable blood pressure and temperature during the operation, and patients with excessive blood loss can be treated with blood transfusion. After the operation, all patients were admitted to the neuro-intensive care unit. Their vital signs and consciousness levels were closely monitored. General treatments such as airway management, blood pressure control, body temperature regulation, blood sugar and sodium levels management, and nutritional support were carried out. All patients received labs/ECG/blood gases every day in neuro-intensive care unit. Additionally, specific treatments for improving cerebral blood circulation and neuroprotection were also implemented. After the condition stabilizes, start rehabilitation treatments such as hyperbaric oxygen therapy as soon as possible. Cranioplasty was performed at 6 to 12 weeks after the operation.

### Mild hypothermia therapy

For all patients in the DHC + MH group, mild hypothermia (rectum core monitoring, 35 °C) was induced immediately after the operation via an external cooling device (MTRE Advanced Technologies, Ltd., Israel). According to the present study, we choose 35 °C as target temperature (29). The target temperature of 35 °C was reached within 4–6 h, and the patients were then rewarmed slowly at a rate of no greater than 0.5 °C every 4 h for 8–12 h. The equipment automatically adjusts according to the target core body temperature. All patients were cooled for 5 to 7 days. All mild hypothermia patients received sedation and analgesia (dexmedetomidine and remifentanil) during the intensive care unit (ICU) period. In the DHC + MH group, the drugs used for mild hypothermia included dexmedetomidine (interquartile range, 0.3–0.5 μg/kg per h), meperidine (8.0–10.0 mg/h), and chlorpromazine hydrochloride (50–100 mg/d). The depth of sedation depends on the bispectral index (BIS) value, the ideal range of BIS was 30 to 50. In the treatment of patients with complete MCA infarction, assisted hypothermia employs multimodal brain monitoring, which includes measuring the ICP, cerebral perfusion pressure (CPP), and BIS (25). All patients underwent dynamic electrocardiography monitoring, and coagulation mechanism monitoring every day, blood gas analysis was evaluated every 2 h. If the patient experiences shivering, it indicates that the optimal level of sedation and analgesia is not sufficient. Therefore, the depth of sedation and analgesia should be adjusted (Appropriately increase the dosage of sedative and analgesic drugs).

### Perioperative management

Every patient underwent early hemicraniectomy within 48 h of symptom onset and was cared for in a specialized neurointensive care unit (NCU) with the best medical treatment available according to the previous studies (24, 30, 31). Perioperative management was planned by two neurosurgeons and one NICU doctor. Patients in both groups underwent the same post-operative treatments, which involved antiepileptics, anti-infective therapy, hypertonic saline, mannitol, sedation, and pain management. All patients had their core body temperature measured via bladder catheters. Vital signs, Multimodal monitoring (such as BIS, TCD, ICP) were monitored. Cranial CT results were routinely examined at 1, 24, and 72 h post-operation if the patients remained stable. The appropriate levels of oxygenation, blood pressure, electrolyte balance, coagulation, and glucose were upheld. All patients need to be closely monitored for complications and proactive measures should be taken to prevent them.

### Statistical methods

Continuous variables are reported as the means with standard deviations or medians with interquartile ranges, and categorical variables are described using frequencies and percentages. Statistical analyses were conducted via SPSS Version 14.0 (SPSS Inc., Chicago, Illinois, USA). *t*-test/Wilcoxon was used for continuous. The χ^2^ test or Fisher exact test was used for categorical variables. All variables according to clinical implications were entered into the multivariate logistic regression. The analysis of post-operative complications, to control for multiple comparisons across the 14 complications, we applied both Bonferroni correction (α = 0.05/14 = 0.00357) and the Benjamini-Hochberg procedure (*FDR* = 0.05). The findings should be interpreted as hypothesis-generating and require validation in independent cohorts. A *P*-value < 0.05 was considered to indicate statistical significance.

## Results

### Clinical data

Between January 2017 and January 2023, 624 patients who were diagnosed with complete middle cerebral artery infarction were treated at our hospital; 119 patients underwent DHC + MH, and 91 patients underwent DHC surgery alone. The baseline data for both groups were not significantly different (*P* > 0.05, Table 2). In the DHC + MH group, 44 (37.0%) patients underwent intravenous thrombolysis, and 22 (18.5%) patients underwent mechanical thrombectomy. There were 27 (29.7%) patients who underwent intravenous thrombolysis and 18 (19.8%) who underwent mechanical thrombectomy in the DHC surgery alone group.

**Table 2:** Clinical baseline of two group.

Patient characteristics	DHC + MH	DHC	*P*-value
No. patients	119	91	
Mean Age (years, mean ± SD)	63.8 ± 12.5	66.6 ± 11.7	0.108
Sex, *n*, 100%			
Male	65 (54.6%)	51 (56.0%)	
Female	54 (45.4%)	40 (44.0%)	
Admitted NIHSS total score (mean ± SD)	16.7 ± 6.5	16.3 ± 6.8	0.675
Admitted GCS (mean ± SD)	7.7 ± 2.9	7.6 ± 3.3	0.844
Comorbidities, *n*, 100%			
Hypertension	73 (61.3%)	59 (64.8%)	0.604
Hyperlipidemia	57 (47.9%)	42 (46.2%)	0.802
Smoking	46 (38.7%)	29 (31.9%)	0.309
Diabetes	34 (28.6%)	31 (34.1%)	0.393
Atrial fibrillation	30 (25.2%)	16 (17.6%)	0.185
Prior stroke(s)	21 (17.6%)	17 (18.7%)	0.847
TOAST classification, *n*, 100%			
Large artery atherosclerosis	68 (57.1%)	47 (51.6%)	0.428
Undetermined etiology	25 (21.0%)	23 (25.3%)	0.466
Cardioembolism	26 (21.9%)	21 (23.1%)	0.832
Acute intervention, *n*, 100%			
Intravenous thrombolysis	44 (37.0%)	27 (29.7%)	0.268
Mechanical thrombectomy	22 (18.5%)	18 (19.8%)	0.813
Arterial recanalization successful, *n*, 100%	108 (90.8%)	80 (87.9%)	0.505
Admitted to DHC (h, mean ± SD)	29.9 ± 13.2	29.0 ± 16.3	0.636
MLS (mm, mean ± SD)	10.0 ± 3.0	10.7 ± 3.5	0.120
Pupillary dilation pre-operation	40 (33.6%)	27 (29.7%)	0.544

NIHSS, National Institutes of Health Stroke Scale; GCS, glasgow coma scale; DHC, decompressive hemicraniectomy; MH, mild hypothermia; MLS, peak midline shift; SD, standard deviation.

After multivariate logistic regression, we found that NIHSS scores (*OR* = 1.196, 95% CI: 1.070–1.336, *P* = 0.002, Table 3), Diabetes (*OR* = 3.685, 95% CI: 1.090–12.456, *P* = 0.036, Table 3), Admitted to DHC time (*OR* = 1.186, 95% CI: 1.113–1.264, *P* = 0.000, Table 3), and Pupillary dilation pre-operation (*OR* = 9.439, 95% CI: 1.319–67.558, *P* = 0.025, Table 3) have an impact on the patient's prognosis. The presence of symptoms Diabetes or Pupillary dilation pre-operation indicates a greater risk of poor prognosis. While lower NIHSS scores, and early DHC may be more beneficial for the patient's prognosis.

**Table 3:** Multivariate regression analysis for outcome in all patients.

Characteristics	B	S.E	Wals	Sig.	Exp (B)	EXP (B) 95% C.I.	
						Down	Up
Sex	−0.336	0.483	0.483	0.487	0.715	0.277	1.843
Age	−0.071	0.058	1.493	0.222	0.932	0.831	1.044
NIHSS	0.179	0.057	9.936	0.002	1.196	1.070	1.336
GCS score	0.187	0.217	0.749	0.387	1.206	0.789	1.844
Hypertension	0.503	0.588	0.732	0.392	1.654	0.522	5.243
Hyperlipidemia	0.047	0.492	0.009	0.924	1.048	0.400	2.748
Smoking	−0.534	0.531	1.014	0.314	0.586	0.207	1.658
Diabetes	1.304	0.621	4.405	0.036	3.685	1.090	12.456
Atrial fibrillation	0.048	0.603	0.006	0.937	1.049	0.322	3.419
Prior stroke(s)	−0.401	0.759	0.279	0.597	0.670	0.151	2.966
Intravenous thrombolysis	0.334	0.676	0.244	0.622	1.396	0.371	5.252
Mechanical thrombectomy	−0.408	0.768	0.283	0.595	0.665	0.148	2.995
Admitted to DHC time	0.171	0.032	27.829	0.000	1.186	1.113	1.264
MLS	0.148	0.126	1.379	0.240	1.159	0.906	1.484
Pupillary dilation pre-operation	2.245	1.004	4.997	0.025	9.439	1.319	67.558

NIHSS, National Institutes of Health Stroke Scale; GCS, Glasgow Coma Scale; DHC, decompressive hemicraniectomy; MH, mild hypothermia; MLS, peak midline shift; SD, standard deviation; CI, confidence intervals; OR, odds ratios.

### Clinical outcome: 6-month mRS score and 30-day all-cause mortality

**Figure 2:**
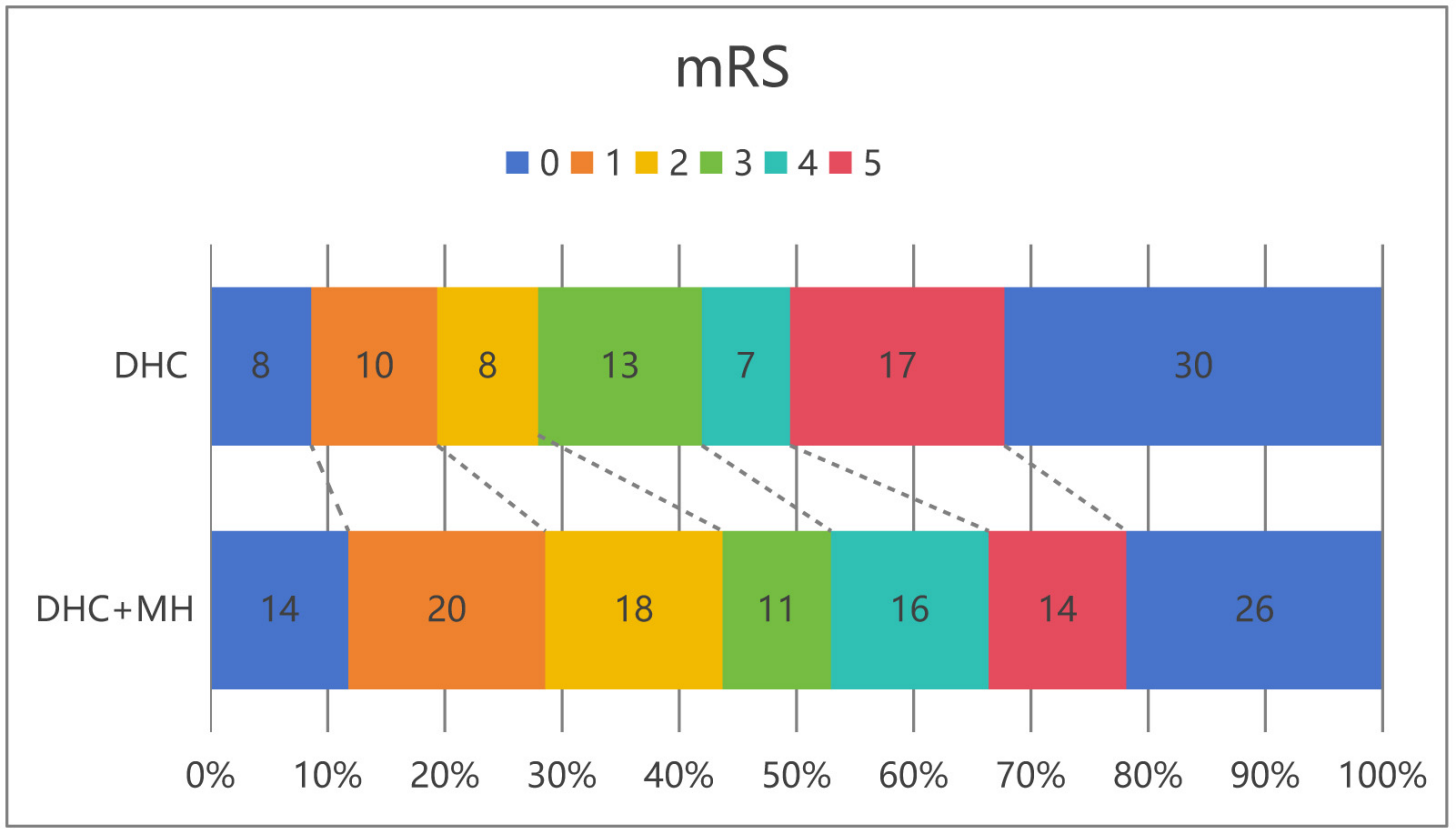
Distribution of 6-month modified Rankin scale scores in patients receiving DHC alone vs. those receiving DHC combined with mild hypothermia.

Six months post-operation, the neurologic outcome was evaluated by two neurosurgeons via mRS scores obtained through either a telephone consultation or an in-person interview. Contact was made with all 210 patients, and follow-up procedures for survivors and the handling of deaths in the analysis set. Fifty-two (43.7%) patients in the DHC + MH group had good outcomes with mRS scores ranging from 0–2, and 26 (28.6%) patients in the DHC group had good outcomes with mRS scores ranging from 0–2. There was a notable difference in the mRS score between the two groups (*OR* = 1.940, 95% CI: 1.085–3.470, *P* = 0.025, Table 4). We also observed 30-day all-cause mortality in the two groups; 21 (17.6%) patients died in the DHC + MH group, and 27 (29.7%) patients died in the DHC group (*OR* = 0.508, 95% CI: 0.265–0.974, *P* = 0.04). This included patients who died within 30 days and not included patients whose family members stopped treatment. The main causes of death were uncontrollable malignant intracranial hypertension, sepsis and pneumonia (Figure 2, Table 4).

**Table 4:** Clinical outcome of two group.

Characteristics	DHC + MH	DHC	95% CI	*OR*	*P*-value
No. patients	119	91			
mRS			1.085–3.470	1.940	0.025
Good (0–2)	52 (43.7%)	26 (28.6%)			
Poor (3–6)	67 (56.3%)	65 (71.4%)			
30-day all cause mortality	21 (17.6%)	27 (29.7%)	0.265–0.974	0.508	0.04

mRS, modified Rankin Scale; DHC, decompressive hemicraniectomy; MH, mild hypothermia; CI, confidence intervals; OR, odds ratios.

### Complications

We also evaluated the degree of complications between the two groups and evaluated the safety of DHC + MH. The most common complications were bBradycardia (68.1%), pneumonia (66.4%) and electrolyte disturbance (65.5%) in the DHC + MH group. Most of these values were greater than those in the DHC alone group. However, most of these methods are not clinically important, such as shivering, bradycardia, acidosis, controllable electrolytic disturbances or hypo- or hypertension. Shivering (46.2% vs. 6.6%, *OR* = 12.174, 95% CI: 4.935–30.033, *P* = 0.000), bradycardia (68.1% vs. 38.5%, *OR* = 3.411, 95% CI: 1.925–6.041, *P* = 0.000), electrolyte disturbance (65.5% vs. 38.5%, *OR* = 3.044, 95% CI: 1.727–5.365, *P* = 0.000), and acidosis (35.5% vs. 18.7%, *OR* = 2.374, 95% CI: 1.243–4.537, *P* = 0.008) occurred significantly more frequently in the DHC + MH group. There was no difference in the incidence of delayed intracranial hematomas, pneumonia, AKI or coagulation disorders, which are very important for clinical outcomes. Other complications, including tachycardia, hypotension, hypertension, hypoxemia, hypercapnia, and alkalosis, were not significantly different between the two groups. After Bonferroni correction, shivering, bradycardia, and electrolyte disturbance remained significantly more frequent in the DHC + MH group (all *P* < 0.001). Acidosis was no longer significant (*P* = 0.008, corrected threshold α = 0.00357). Using the less conservative *FDR* approach, acidosis was also considered significant (*P* = 0.008 < 0.0143). All other complications showed no significant intergroup differences after correction (Table 5).

**Table 5:** Complications between two group.

Characteristics	DHC + MH	DHC	*P*-value	Bonferroni correction	BH (*FDR*)
No. patients	119	91			
Complications					
Delayed intracranial hematomas	25 (21.0%)	22 (24.2%)	0.585	1.000	0.819
Pneumonia	79 (66.4%)	52 (57.1%)	0.171	1.000	0.239
AKI	16 (13.4%)	9 (9.9%)	0.430	1.000	0.602
Shivering	55 (46.2%)	6 (6.6%)	0.000^*^	0.000^*^	0.000^*^
Bradycardia	81 (68.1%)	35 (38.5%)	0.000^*^	0.000^*^	0.000^*^
Tachycardia	25 (21%)	13 (14.3%)	0.268	1.000	0.375
Hypotension	46 (38.7%)	30 (33.0%)	0.395	1.000	0.553
Hypertension	33 (27.7%)	28 (30.8%)	0.631	1.000	0.883
Coagulation disorders	18 (15.1%)	7 (7.7%)	0.099	1.000	0.138
Blood gas analysis					
Hypoxemia	53 (44.5%)	31 (34.1%)	0.125	1.000	0.175
Hypercapnia	27 (22.7%)	14 (15.4%)	0.186	1.000	0.175
Alkalosis	46 (38.7%)	34 (37.4)	0.848	1.000	1.000
Acidosis	42 (35.5%)	17 (18.7%)	0.008^*^	0.112	0.011^*^
Electrolyte disturbance	78 (65.5%)	35 (38.5%)	0.000^*^	0.000^*^	0.000^*^

AKI, acute kidney injury; DHC, decompressive hemicraniectomy; MH, mild hypothermia; FDR: Benjamini-Hochberg.

^*^Statistically significant (*P* < 0.05).

## Discussion

We retrospectively reviewed the clinical outcomes and complications of patients who underwent DHC + MH or DHC alone by analyzing a 6-month, three-center, consecutive patient cohort in China. In the present study, a total of 210 cases of DHC among 4,250 acute ischemic stroke patients were reported, which means that 4.5% of acute ischemic patients underwent DHC at three Chinese hospitals. As some DHC cases were excluded by the exclusion criteria, the real rate of DHC may be higher than 4.5%. This percentage was higher than that reported in previous studies in Japan (0.73%) (32) and the United States (0.07%) (33), possibly because most severe acute ischemic stroke patients were admitted to our three hospitals, as these hospitals are advanced comprehensive stroke centers. Decompressive craniectomy is a widely used and life-saving surgical treatment for patients with uncontrollable intracranial hypertension resulting from traumatic brain injuries, acute ischemic stroke, and intracranial hematoma (8, 9, 12, 34). However, decompressive craniectomy alone still does not control malignant intracranial hypertension in some cases (25).

Mild hypothermia has been shown to have a beneficial effect on animal MCA infarction experiments (19, 35–37). Doerfler (35) reported that early DHC can significantly reduce the infarction size and improve neurological outcomes and that the combination of DHC with mild hypothermia significantly enhances the neuroprotective benefit in MCA infarction experiments in rats. Su (38) reported that in a randomized controlled trial involving 16 patients in the hypothermia (33 or 34 °C) group and 17 patients in the control group (ChiCTR-TCS-12002680), mild hypothermia may improve neurological outcomes in living patients, even though it cannot reduce mortality in patients with massive cerebral hemispheric infarction. Piironen (21) also reported in a trial involving mild hypothermia for awake stroke patients after intravenous thrombolysis (NCT00987922), 36 patients were enrolled and randomly divided into two groups: one receiving mild hypothermia at 35 °C and the other receiving standard stroke unit care, all within 6 h of symptom onset. The target temperature of 35 °C used in this study may represent strict temperature control rather than conventional therapeutic hypothermia. Additionally, Jeong reported that mild hypothermia at 35 °C after successful recanalization via endovascular treatment in acute ischemic stroke is safe and feasible, despite some adverse events (29). The study also revealed that mild hypothermia is a safe and practical option for stroke patients undergoing thrombolysis and breathing on their own, even with some adverse events. Hong (39) also evaluated another interesting study on mild hypothermia and reported that it could be beneficial for acute ischemic stroke patients after recanalization by potentially reducing cerebral edema and hemorrhagic transformation risk and improving clinical outcomes. They also randomized 39 patients in the hypothermia group and 36 patients in the normothermia group. However, contrary to the results of a recent randomized clinical trial on the outcomes of hypothermia combined with DHC in patients with malignant MCA stroke (DRKS00000623), 24 patients were randomized to standard care, and 26 patients were randomized to moderate hypothermia (33.0 ± 1.0 °C). The results demonstrated that early moderate hypothermia after DHC is not associated with favorable functional outcomes compared with standard care. In contrast, moderate hypothermia may cause serious harm in moderate hypothermia patients (40). All four of these randomized clinical trials had a common severe limitation, which was the small sample size, which might not allow the detection of significant differences between two groups and a lower quality of evidence. In the present study, we found that DHC combined with mild hypothermia was associated with favorable 6-month outcomes (mRS scores) and lower 30-day all-cause mortality, but these findings should be interpreted with caution due to potential selection bias and unmeasured confounding. Further randomized controlled trials are needed to confirm causality. Additionally, in terms of patient selection, the patients in the MH treatment group might have more severe conditions, and after decompressive craniectomy, the intracranial pressure might be even higher.

Many previous studies have reported that DHC + MH in patients with malignant brain infarction or severe traumatic brain injury were not associated with favorable functional outcomes because of severe side effects, including bradycardia, pneumonia, electrolyte disturbance, shivering, acidosis, AKI and coagulation disorders (41–45). Differences in studies, such as the length and intensity of hypothermia, might affect their clinical effectiveness. With the progress of neural monitoring technology, including multimodal brain monitoring and more effective perioperative management (24, 25, 46), mild hypothermia-related complications have become controllable. In the present study, there were no significant differences in mild hypothermia-related complications, including delayed intracranial hematomas, pneumonia, acute kidney injury, coagulation disorders, etc. In addition to shivering (*P* = 0.000), bradycardia (*P* = 0.000), acidosis (*P* = 0.008), and electrolyte disturbance (*P* = 0.002), these complications were almost always temporary. Good monitoring, prevention and treatment of complications generally do not affect the prognosis of patients. As the substantially higher rate of hypothermia-associated complications, it requires us to pay close attention in clinic. The following suggestions are recommended when mild hypothermia is performed: Multimodal brain monitoring, including ICP monitoring, CPP, transcranial Doppler (TCD) ultrasonography and BIS, is needed as much as possible, and monitoring vital signs, blood gas analysis, coagulation, etc., is needed more often to minimize related complications; analgesia and sedation are very important for DHC + MH patients; long-term mild hypothermia lasting 5–7 days is more effective than short-term mild hypothermia of 24–48 h for managing refractory intracranial hypertension or enhancing neurological outcomes in severe traumatic brain injury or cardiac arrest (47–49); and rewarming at a slow pace with 0.5 °C every 4 h for 8–12 h. Rapid rewarming may cause intracranial pressure to rebound and lead to poor outcomes. Kaneko (50) also reported that a randomized controlled trial suggested that slow rewarming for more than 48 h could enhance neurological outcomes in patients with TBI and an evacuated hematoma undergoing prolonged mild therapeutic hypothermia. The potential effects of rewarming on patient outcomes were similar in experimental shock (51) patients and in poor-grade subarachnoid hemorrhage patients (52).

This study has several limitations. The main limitation is its retrospective nature, our study is subject to potential selection bias because treatment allocation was not randomized and was influenced by physician judgment, patient preference, and economic considerations. Second, the study was performed at a single center. Third, a robust statistical examination was conducted, starting with univariate analysis and followed by multivariate regression analysis, considering all confounding factors. Thus, prospective randomized controlled trials with larger sample sizes are needed to further confirm the outcome and safety of DHC + MH in patients with malignant MCA infarction.

## Conclusion

In this retrospective multi-center cohort, mild hypothermia during decompressive hemicraniectomy was associated with favorable unadjusted outcomes. Given non-randomized allocation and potential confounding, these findings are hypothesis-generating and require confirmation with adjusted analyses and prospective evaluation. The procedure needs to be validated in larger, multicenter, prospective, randomized controlled trials.

## Data Availability

The raw data supporting the conclusions of this article will be made available by the authors, without undue reservation.

## References

[1] Feigin VL Nguyen G Cercy K Johnson CO Alam T Parmar PG . Global, regional, and country-specific lifetime risks of stroke, 1990 and 2016. New England J Med. (2018) 379:2429–37. doi: 10.1056/NEJMoa1804492 30575491 PMC6247346

[2] Allen C Biryukov S Giussani G Gugnani H Hay SI Millear A . Global, regional, and national disability-adjusted life-years (DALYs) for 315 diseases and injuries and healthy life expectancy (HALE), 1990-2015: a systematic analysis for the global burden of disease study. (2016). Lancet. 388:1603. doi: 10.1016/S0140-6736(16)31460-X PMC5388857 27733283

[3] Lallukka T Millear A Pain A Cortinovis M Giussani G . Global, regional, and national life expectancy, all-cause mortality, and cause-specifi c mortality for 249 causes of death, 1980-2015: a systematic analysis for the global burden of disease study 2015. (2017). Lancet. 388:1459. doi: 10.1016/S0140-6736(16)31012-1 PMC5388903 27733281

[4] Daou B Kent AP Montano M Chalouhi N Starke RM Tjoumakaris S . Decompressive hemicraniectomy: predictors of functional outcome in patients with ischemic stroke. J Neurosurg. (2016) 124:1773–9. doi: 10.3171/2015.6.JNS15729 26613165

[5] Huttner HB Schwab S. Malignant middle cerebral artery infarction: clinical characteristics, treatment strategies, and future perspectives. Lancet Neurol. (2009) 8:949–58. doi: 10.1016/S1474-4422(09)70224-8 19747656

[6] White OB Norris JW Hachinski VC Lewis A. Death in early stroke, causes and mechanisms. Stroke. (1979) 10:743–743. doi: 10.1161/01.STR.10.6.743 524417

[7] Goedemans T Verbaan D Coert BA Kerklaan BJ Van Den Berg R Coutinho JM . Neurologic outcome after decompressive craniectomy: predictors of outcome in different pathologic conditions. World Neurosurg. (2017) 105:765–74. doi: 10.1016/j.wneu.2017.06.069 28642178

[8] Walz B Zimmermann C Bottger S Haberl RL. Prognosis of patients after hemicraniectomy in malignant middle cerebral artery infarction. J Neurol. (2002) 249:1183–90. doi: 10.1007/s00415-002-0798-x 12242536

[9] Powers WJ Rabinstein AA Ackerson T Adeoye OM Bambakidis NC Becker K . Guidelines for the early management of patients with acute ischemic stroke: a guideline for healthcare professionals from the American heart association/american stroke association. Stroke. (2018) 49:E46–E110. doi: 10.1161/STR.0000000000000158 29367334

[10] Hofmeijer J Kappelle LJ Algra A Amelink GJ Van Gijn J Van Der Worp HB . Surgical decompression for space-occupying cerebral infarction (the hemicraniectomy after middle cerebral artery infarction with life-threatening edema trial hamlet): a multicentre, open, randomised trial. Lancet Neurol. (2009) 8:326–33. doi: 10.1016/S1474-4422(09)70047-X 19269254

[11] Frank JI Schumm LP Wroblewski K Chyatte D Rosengart AJ Kordeck C . Hemicraniectomy and durotomy upon deterioration from infarction-related swelling trial randomized pilot clinical trial. Stroke. (2014) 45:781–7. doi: 10.1161/STROKEAHA.113.003200 24425122 PMC4033520

[12] Juettler E Unterberg A Woitzik J Boesel J Amiri H Sakowitz OW . Hemicraniectomy in older patients with extensive middle-cerebral-artery stroke. New England J Med. (2014) 370:1091–100. doi: 10.1056/NEJMoa1311367 24645942

[13] Staykov D Gupta R. Hemicraniectomy in malignant middle cerebral artery infarction. Stroke. (2011) 42:513–6. doi: 10.1161/STROKEAHA.110.605642 21212397

[14] Rahme R Curry R Kleindorfer D Khoury JC Ringer AJ Kissela BM . How often are patients with ischemic stroke eligible for decompressive hemicraniectomy? Stroke. (2012) 43:550–2. doi: 10.1161/STROKEAHA.111.635185 22034001 PMC3265663

[15] Basu P Jenkins H Tsang K Vakharia VN. National survey of neurosurgeons and stroke physicians on decompressive hemicraniectomy for malignant middle cerebral artery infarction. World Neurosurg. (2017) 102:320–8. doi: 10.1016/j.wneu.2017.02.043 28235642

[16] Fehnel CR Lee Y Wendell LC Thompson BB Potter NS Mor V. Utilization of long-term care after decompressive hemicraniectomy for severe stroke among older patients. Aging Clin Exp Res. (2017) 29:631–8. doi: 10.1007/s40520-016-0615-5 27495258

[17] Sun Y-J Zhang Z-Y Fan B Li G-Y. Neuroprotection by therapeutic hypothermia. Front Neurosci. (2019) 13. doi: 10.3389/fnins.2019.00586 31244597 PMC6579927

[18] Tu Y Guo C Song F Huo Y Geng Y Guo M . Mild hypothermia alleviates diabetes aggravated cerebral ischemic injury via activating autophagy and inhibiting pyroptosis. Brain Res Bull. (2019) 150:1–12. doi: 10.1016/j.brainresbull.2019.05.003 31082455

[19] Oyama Y Ono K Kawamura MJr. Mild hypothermia protects synaptic transmission from experimental ischemia through reduction in the function of nucleoside transporters in the mouse hippocampus. Neuropharmacology. (2020) 163:107853–107853. doi: 10.1016/j.neuropharm.2019.107853 31734385

[20] Schwab S Georgiadis D Berrouschot J Schellinger PD Graffagnino C Mayer SA. Feasibility and safety of moderate hypothermia after massive hemispheric infarction. Stroke. (2001) 32:2033–5. doi: 10.1161/hs0901.095394 11546893

[21] Piironen K Tiainen M Mustanoja S Kaukonen K-M Meretoja A Tatlisumak T . Mild hypothermia after intravenous thrombolysis in patients with acute stroke a randomized controlled trial. Stroke. (2014) 45:486–91. doi: 10.1161/STROKEAHA.113.003180 24436240

[22] Schwab S Schwarz S Spranger M Keller E Bertram M Hacke W. Moderate hypothermia in the treatment of patients with severe middle cerebral artery infarction. Stroke. (1998) 29:2461–6. doi: 10.1161/01.STR.29.12.2461 9836751

[23] Els T Oehm E Voigt S Klisch J Hetzel A Kassubek J. Safety and therapeutical benefit of hemicraniectomy combined with mild hypothermia in comparison with hemicraniectomy alone in patients with malignant ischemic stroke. Cerebrovasc Dis. (2006) 21:79–85. doi: 10.1159/000090007 16330868

[24] Chen J-H Li P-P Yang K Chen L Zhu J Hu X . Value of ventricular intracranial pressure monitoring for traumatic bifrontal contusions. World Neurosurg. (2018) 113:E690–701. doi: 10.1016/j.wneu.2018.02.122 29501515

[25] Chen J-H Xu Y-N Ji M Li P-P Yang L-K Wang Y-H. Multimodal monitoring combined with hypothermia for the management of severe traumatic brain injury: a case report. Exp Ther Med. (2018) 15:4253–8. doi: 10.3892/etm.2018.5994 29731820 PMC5921228

[26] Vahedi K Hofmeijer J Juettler E Vicaut E George B Algra A . Early decompressive surgery in malignant infarction of the middle cerebral artery: a pooled analysis of three randomised controlled trials. Lancet Neurol. (2007) 6:215–22. doi: 10.1016/S1474-4422(07)70036-4 17303527

[27] Zhang LM Li R Zhao XC Wang ML Fu Y. The relationship between colloid transfusion during surgical decompression hemicraniectomy period and postoperative pneumonia or long-term outcome after space-occupying cerebral infarction: a retrospective study. World Neurosurg. (2019) 122:E1312–20. doi: 10.1016/j.wneu.2018.11.041 30448584

[28] Delashaw JB Broaddus WC Kassell NF Haley EC Pendleton GA Vollmer DG . Treatment of right hemispheric cerebral infarction by hemicraniectomy. Stroke. (1990) 21:874–81. doi: 10.1161/01.STR.21.6.874 2349590

[29] Jin-Heon J Jeong-Ho H Sung-Il S Hyungjong P Jun Young C Kyu Sun Y . Mild hypothermia after endovascular treatment for acute ischemic stroke: a pilot. Randomized Controlled Trial. (2025) 56: 3100–7. doi: 10.1161/STROKEAHA.124.049762 40791183

[30] George N., Tomasz, D., Patrik, M., Vasileios, P., Jesper, P., Dimitre, S., et al. (2015). European Stroke Organisation (ESO) guidelines for the management of temperature in patients with acute ischemic *stroke*. 10: 941–9. doi: 10.1111/ijs.12579 26148223

[31] Hermann N Hauke S Julian B Carsten H Sven P Rainer K . Outcomes of hypothermia in addition to decompressive hemicraniectomy in treatment of malignant middle cerebral artery stroke: a randomized clinical Trial. (2019) JAMA Neurology. 76:571–9. 30657812 10.1001/jamaneurol.2018.4822 PMC6515837

[32] Suyama K Horie N Hayashi K Nagata I. Nationwide survey of decompressive hemicraniectomy for malignant middle cerebral artery infarction in Japan. World Neurosurg. (2014) 82. doi: 10.1016/j.wneu.2014.07.015 25045787

[33] Adeoye O Hornung R Khatri P Ringer A Kleindorfer D. The rate of hemicraniectomy for acute ischemic stroke is increasing in the United States. J Stroke Cerebr Dis. (2011) 20:251–4. doi: 10.1016/j.jstrokecerebrovasdis.2010.01.006 20621514

[34] Beez T Munoz-Bendix C Steiger H-J Beseoglu K. Decompressive craniectomy for acute ischemic stroke. Critical Care. (2019) 23. doi: 10.1186/s13054-019-2490-x 31174580 PMC6556035

[35] Doerfler A Schwab S Hoffmann TT Engelhorn T Forsting M. Combination of decompressive craniectomy and mild hypothermia ameliorates infarction volume after permanent focal ischemia in rats. Stroke. (2001) 32:2675–81. doi: 10.1161/hs1101.098369 11692033

[36] Bian JY Zhong W Zhang SM Dai Z Kato Y Kanno T . Decompressive craniectomy and mild hypothermia reduces infarction size and counterregulates Bax and Bcl-2 expression after permanent focal ischemia in rats. Neurosurg Rev. (2006) 29:168–72. doi: 10.1007/s10143-005-0010-8 16402275

[37] Lee JH Wei L Gu X Won S Wei ZZ Dix TA . Improved therapeutic benefits by combining physical cooling with pharmacological hypothermia after severe stroke in rats. Stroke. (2016) 47:1907–13. doi: 10.1161/STROKEAHA.116.013061 27301934 PMC4927220

[38] Su YY Fan LL Zhang YZ Zhang Y Ye H Gao DQ . Improved neurological outcome with mild hypothermia in surviving patients with massive cerebral hemispheric infarction. Stroke. 47:457. doi: 10.1161/STROKEAHA.115.009789 26696645

[39] Hong JM Lee JS Song H-J Jeong HS Choi HA Lee K. Therapeutic hypothermia after recanalization in patients with acute ischemic stroke. Stroke. (2014) 45:134–40. doi: 10.1161/STROKEAHA.113.003143 24203846

[40] Neugebauer H., Schneider, H., and Bosel, J. (2019). Outcomes of hypothermia in addition to decompressive hemicraniectomy in treatment of malignant middle cerebral artery stroke: a randomized clinical trial. Jama Neurology 76:626. doi: 10.1001/jamaneurol.2018.4822 PMC6515837 30657812

[41] Polderman KH. Application of therapeutic hypothermia in the ICU: opportunities and pitfalls of a promising treatment modality. Part 1: indications and evidence intensive care. Medicine. 30:556–75. doi: 10.1007/s00134-003-2152-x 14767591

[42] Hutchison JS Ward RE Lacroix J Hebert PC Barnes MA Bohn DJ . Hypothermia therapy after traumatic brain injury in children. New England J Med. (2008) 358:2447–56. doi: 10.1056/NEJMoa0706930 18525042

[43] Clifton GL Valadka A Zygun D Coffey CS Drever P Fourwinds S . Very early hypothermia induction in patients with severe brain injury (the national acute brain injury study: hypothermia II): a randomised trial. Lancet Neurol. (2011) 10:131–9. doi: 10.1016/S1474-4422(10)70300-8 21169065 PMC3628679

[44] Urbano LA Oddo M. Therapeutic hypothermia for traumatic brain injury. Curr Neurol Neurosci Rep. (2012) 12:580–91. doi: 10.1007/s11910-012-0304-5 22836524

[45] Nielsen N Wettersley J Cronberg T Erlinge D Gasche Y Hassager C . Targeted temperature management at 33°C versus 36°C after cardiac arrest. New England J Med. (2013) 369:2197–206. doi: 10.1056/NEJMoa1310519 24237006

[46] He J Chen J Wu T Zhang C Yang L Shi Z-H . The value of managing severe traumatic brain injury during the perioperative period using intracranial pressure monitoring. J Craniof Surg. (2019) 30:2217–23. doi: 10.1097/SCS.0000000000005861 31469742

[47] Jiang JY Yu MK Zhu C. Effect of long-term mild hypothermia therapy in patients with severe traumatic brain injury: 1-year follow-up review of 87 cases. J Neurosurg. (2000) 93:546–9. doi: 10.3171/jns.2000.93.4.0546 11014530

[48] Jiang JY Xu W Li WP Gao GY Bao YH Liang YM . Effect of long-term mild hypothermia or short-term mild hypothermia on outcome of patients with severe traumatic brain injury. Journal of Cerebral Blood Flow and Metabolism. (2006) 26:771–6. doi: 10.1038/sj.jcbfm.9600253 16306933

[49] Maynard C Longstreth WTJr Nichol G Hallstrom A Kudenchuk PJ Rea T . Effect of prehospital induction of mild hypothermia on 3-month neurological status and 1-year survival among adults with cardiac arrest: long-term follow-up of a randomized, clinical trial. J Am Heart Assoc. (2015) 4:45–52. doi: 10.1161/JAHA.114.001693 PMC4392445 25762805

[50] Kaneko T Fujita M Yamashita S Oda Y Suehiro E Dohi K . Slow rewarming improved the neurological outcomes of prolonged mild therapeutic hypothermia in patients with severe traumatic brain injury and an evacuated hematoma. Sci Rep. (2018) 8. doi: 10.1038/s41598-018-30119-z 30072782 PMC6072739

[51] Burggraf M Lendemans S Waack IN Teloh JK Effenberger-Neidnicht K Jaeger M . Slow as Compared to Rapid Rewarming After Mild Hypothermia Improves Survival in Experimental Shock. J Surg Res. (2019) 236:300–10. doi: 10.1016/j.jss.2018.11.057 30694770

[52] Choi W Kwon SC Lee WJ Weon YC Choi B Lee H . Feasibility and safety of mild therapeutic hypothermia in poor-grade subarachnoid hemorrhage: prospective pilot study. J Korean Med Sci. (2017) 32:1337–44. doi: 10.3346/jkms.2017.32.8.1337 28665071 PMC5494334

